# Production of a potential liquid plant bio-stimulant by immobilized *Piriformospora indica* in repeated-batch fermentation process

**DOI:** 10.1186/s13568-017-0408-z

**Published:** 2017-05-25

**Authors:** Nikolay Vassilev, Bettina Eichler-Löbermann, Elena Flor-Peregrin, Vanessa Martos, Antonia Reyes, Maria Vassileva

**Affiliations:** 1 Institute of Biotechnology, University of Granada, c/Fuentenueva s/n, 18071 Granada, Spain; 2 Institute for Land Use, University of Rostock, Universitätsplatz 1, 18055 Rostock, Germany; 3 Department of Plant Physiology, University of Granada, c/Fuentenueva s/n, 18071 Granada, Spain

**Keywords:** Biofertilizers, *Piriformospora indica*, Immobilization, P solubilization, Plant growth promotion

## Abstract

*Piriformospora indica*, a mycorrhizal-like fungus able to establish associations with roots of a wide range of plants, supporting plant nutrition and increasing plant resistance and tolerance to stress, was shown to solubilise phosphate applied in the form of animal bone char (HABO) in fermentation systems. The process of P solubilisation was caused most likely by proton extrusion and medium pH lowering. The fungal mycelium was successfully immobilized/retained in a polyurethane foam carrier. Further employment of the immobilized mycelium in repeated-batch fermentation process resulted in at least 5 cycles of P solubilization. The concentration of soluble P increased during the experiment with 1.0 and 3.0 g HABO l^−1^ and at the end of the 5th batch cycle reached 40.8 and 120 mg l^−1^, respectively. The resulting final liquid product, without or with solubilized phosphate, was found to significantly increase plant growth and P plant uptake. It can be used as a biostimulant containing microbial plant growth-promoting substances and soluble P derived from renewable sources (HABO) thus supporting the development of sustainable agro-ecosystems.

## Introduction

Chemical fertilizers and pesticides are used for fertilizing crops and controlling pests thus successfully increasing production capacity of farm systems. However, the massive and long-term use of these chemical products has negatively affected the environment (soil quality/yielding capacity, biodiversity, underground water) and public health (Hazell and Wood 2008). The practice of intensive production methods and indiscriminate application of agrochemicals also provoked a decrease in plant resistance to biotic and abiotic stress factors. One of the most attractive alternatives of the intensive chemical-based production methods that make a positive contribution to environmentally safe sustainable agriculture is the use of plant beneficial microorganisms (Vessey 2003; Gray and Smith 2005; Singh et al. 2011). The beneficial microbial effects include promotion of plant growth, biological control of diseases, increases in crop yield, and quality improvement. In recent years, studies are carried out to produce biofertilizers as efficient formulated products in order to substitute for the chemical fertilizers. Beneficial microbial inoculants in agriculture are mainly plant growth-promoting bacteria and fungi that according to their function are grouped in biofertilizers and biocontrol agents. In fact, they are formulated products containing one or more microorganisms that enhance the nutrient status and health of the plants by either replacing soil nutrients and/or by making nutrients more available to plants and/or by increasing plant access to nutrients or by producing specific metabolites (Malusa and Vassilev 2014). The lack of success of biofertilizers to exert their specific functions reflects problems related with production and formulation of the inocula. The development of a biofertilizer starts with isolation/selection/characterization of an effective microorganism and ends with the main technological steps of the overall biofertilizer production process which are the fermentation mass production process and formulation procedure (Vassilev et al. 2015, 2016). Interestingly, scientific efforts are mainly concentrated on isolation and selection of plant-beneficial microorganisms and their further application in soil–plant system in controlled conditions although immense possibilities exists in developing biotechnological schemes for optimizing/combining mass production and formulation procedure (Vassilev et al. 2015) or direct application of fermentation products avoiding the formulation step.

The most important plant beneficial microorganisms are mycorrhizal fungi and plant-growth promoting microorganisms (*Rhizobium*, P-solubilizing microorganisms, etc.) that manifest different mechanisms to obtain this final effect. Additionally, during the last decade, mycorrhizal-like fungi such as *Piriformospora indica* belonging to *Basidiomycetes* were found to exert different functions including plant growth promotion and P solubilization (Varma et al. 1999). The main advantage of these fungi compared to typical mycorrhizal fungi is their cultivability and rapid production in conventional fermentation systems and easier handling. In some studies, it was also found that filtrate of *P. indica* demonstrated the same effects as spores/mycelium when introduced into soil–plant systems (Bagde et al. 2011; Kumar et al. 2012).

Immobilization of plant beneficial microorganisms is now a well-established biotechnological tool for formulation of inoculants and/or studying their properties in vitro (Vassileva et al. 1998; Vassilev et al. 2005, 2014, 2016). Immobilized microbial cells are characterized by higher metabolic activity and stability (Vassilev and Vassileva 1992), can be used in repeated-batch or continuous fermentations (Vassilev et al. 1990; Kautola et al. 1990), ensure continuous release of cells in soil–plant systems (Vassilev et al. 2001), and form “smart” biofertilizers (Vassilev et al. 2015).

In this work, immobilized *P. indica* was applied in repeated-batch fermentation process in the presence of hydroxyapatite (animal bone char, HABO) to produce a liquid fertilizer with plant-growth properties and enriched with soluble phosphate.

## Materials and methods

### Microorganism

*P. indica* ATCC-204458, used throughout this study, was maintained on *Aspergillus* medium (Hill and Käfer 2001), sub-cultured every 2 months, and incubated at 28 °C for 10 days.

### Fermentation process

Modified Pikovskaya liquid growth medium was used with the following composition (per g l^−1^): glucose, 20.0; NH_4_SO_4_, 1.0; MgSO_4_·7H_2_O, 0.1 g; KCl, 0.2 g; yeast extract, 1.0 g; MnSO_4_·H_2_O, 0.001 g; FeSO_4_·7H_2_O, 0.001 g; pH 7.0 (KOH). Phosphate was applied as 1 g l^−1^ KH_2_PO_4_ in the growth medium and 1.0 or 3.0 g l^−1^ animal bonechar, (BES, Greenock, Scotland; P 14.55%; 20, 60, and 100 mesh), in the Pikovskaya production medium. The composition of the latter medium was the same as the growth medium but with reduced amounts of glucose (10 g l^−1^) and yeast extract (0.25 g l^−1^) and the nitrogen source was NH_4_NO_3_ (0.3 g l^−1^). HABO solubilization by free *P. indica* was carried out by a previously grown 10-day old culture on growth medium. The fungal mycelium was separated from the fermentation liquid, washed with sterile dH_2_O and transferred to 300 ml Erlenmeyer flasks with 100 ml production medium (enriched with HABO).

### *P. indica* passive immobilization in polyurethane foam

The immobilized procedure was as previously described for immobilization of fungi (Kautola et al. 1989). Polyurethane foam (PUF) thoroughly prewashed cubes (0.5 cm^3^; 0.5–0.6 mm pore size) were used as a carrier. The cubes, 0.20–1.0 g flask^−1^, were introduced into 100 ml growth medium in 250-ml Erlenmeyer flasks, which were further loosely capped with cotton and then sterilized in autoclave for 120 °C/20 min. 0.5–5.0 ml of scratched with sterile dH_2_O fungal spores and hyphae were inoculated per flask. Alternatively, two fully colonized circular agar discs (1 cm^2^ from a Petri plate) were inoculated into each Erlenmeyer flask. The flasks were incubated at 28 °C, with constant shaking at 200 rpm on a rotatory shaker (Fisher Scientific). After 10 days of cultivation, the immobilized *P. indica* was separated from the fermentation broth, washed with sterilized distilled water and transferred into 300 ml Erlenmeyer flasks with 100 ml production medium enriched with HABO. At least five batch cycles were carried out in triplicate at 150 rpm, 28 °C, 5 days/single batch.

### Soil–plant experiment

White clover (*Trifolium repens* L. cv. Huia) was selected as the test plant. Before germination, seeds were surface disinfected by immersion in 95% ethanol for 30 s followed by 10 min in 5% hydrogen peroxide (H_2_O_2_) solution. The seeds were then washed thoroughly 5 times with sterile distilled water. The seeds were pre-germinated in Petri dishes with moistened filter paper for 48 h at 25 °C. Three pre-germinated seeds with a root length <1 cm were planted in each pot at 2 cm depth. All experiments were carried out with a steam-sterilized soil–sand mixture (1:1, v/v). Topsoil (0–20 cm) from a field of Granada (Spain) province was used. The main soil characteristics were pH 7.5; 8 µg P per g soil (Olsen test); organic carbon 0.46%; total N 0.046%. The treatments used in this experiment were as follows:

(i) C: Control, dH_2_O was added to each pot of this treatment;
(ii) C+: Control+, half-strength culture medium was added to each pot of this treatment;
(iii) –P: *P. indica* filtrate derived from fermentation process without HABO was added to each pot of this treatment;
(iv) +P: *P. indica*/Pi filtrate derived from fermentation process with HABO (containing solubilized phosphate) was added to each pot of this treatment.

The plants were grown in pots (d = 12.2 cm; 500 g capacity; 4 pots per treatment) in a controlled chamber under a day/night cycle of 16/8 h, 24/17 °C, 50% relative humidity. Humidity was maintained at about 60% field capacity during the experiment. All pots received 1 ml (10^8^ cells ml^−1^) of *Rhizobium trifolii* (Estacion Experimental del Zaidin, CSIC, Spain) grown in 100 ml nutrient broth medium (250 ml Erlenmeyer flasks; pH 6.0; 25 °C; 140 rev min^−1^; 40 h). Each treatment was fertilized with 5 ml of the corresponding solution (C, C+, −P, +P) 3 times/week. Plants were harvested 6 weeks after sowing and analyzed for shoot dry weight, root dry weight, and total shoot P content.

### Analytical methods

Microbial biomass was determined by vacuum filtration of the samples collected at the end of each repeated batch cycle, washed with distilled H_2_O, and then dried at 70 °C to a constant weight. The immobilized biomass in the polyurethane foam cubes was estimated from the difference between the total carrier cell concentration and the initial weight of the carrier. Glucose concentration was measured by enzymatic test combination (R-Biopharm, Darmstadt, Germany). The concentration of soluble phosphate was determined by the molybdo-vanado method using vanadate molybdate reagent (Sigma-Aldrich Cat. No 94686). pH was measured by pH-meter Mettler Toledo FiveEasy F20. All fermentation experiments were performed in triplicate (3 flasks per treatment) and values were analysed and presented as mean ± standard deviation.

Shoot and root dry weights were recorded after drying at 70 °C. Shoot P content was determined by the molybdo-vanado method described by Lachica et al. (1973). Soil–plant experiments were arranged in completely randomized designs with four repetitions. Data were submitted to analysis of variance and treatment means were compared using the Tukey test (P < 0.05).

## Results

### HABO solubilization by free *P. indica*

The aim of the first set of experiments was to test the capacity of the fungal culture to solubilize HABO applied at a concentration of 1.0 g l^−1^. The results showed that *P. indica* grew well in media supplemented with different size of HABO particles (Table 1).

**Table 1:** Solubilization of different particle size of HABO (1.0 g l^−1^) by freely suspended *P. indica* in a single 120-h batch fermentation process

HABO particle size (mesh)	Biomass (mg flask)	Biomass (g g^−1^ sugar)	P_sol_ (mg l^−1^)	P_sol_ yield (% of total P)
C	440.0 ± 0.5	0.69	NA	NA
20	519.9 ± 0.3	0.74	31.0 ± 12	21.3
60	471.4 ± 0.2	0.72	26.1 ± 13	18.0
100	430.4 ± 0.4	0.70	21.9 ± 9	15.1

Values are mean ± SE of three replicates

*NA* not applicable, *P*_*sol*_ soluble phosphate

The biomass grew over the fermentation process, reaching final values ranging from 440.0 mg flask^−1^ in the control treatment without added insoluble phosphate to 519.9 mg flask^−1^ in the treatment supplemented with 20-mesh-HABO. Simultaneously, the fungal culture solubilized the inorganic phosphate, what resulted in 15.1–21.3% solubilization yield (% soluble P of the total amount). The addition of the HABO, its solubilization rate, and the release of phosphate soluble form affected the *P. indica* biomass concentration. The biomass growth and yield (g biomass per gram glucose consumed) was higher when smaller particles (20 mesh) of HABO were used and when the soluble phosphate reached its highest values (31.0 mg l^−1^). When assessing the results of P-solubilizing processes, it is important to note that the pH value and soluble phosphate concentration are amongst the most important factors influencing the overall success of the fermentation process (Vassilev et al. 2012; Mendes et al. 2014). While the increasing phosphate concentration due to the HABO solubilization positively affected the biomass growth, the P-bearing material maintained the pH slightly higher compared to the HABO-free experiments (4.45 and 4.20, respectively). On the other hand, the use of NH_4_NO_3_ as a nitrogen source facilitated the HABO solubilization as *P. indica* did not release organic acids or their concentrations are very low (Ngwene et al. 2016). The compromise between biomass growth/NH_4_NO_3_ consumption determined the results in this trial, as demonstrated in other studies with P-solubilizing microorganisms, which did not produce organic acids (Vassilev et al. 2014).

### *P. indica* immobilization

The next step of this study was to achieve sufficiently stable and functional immobilized particles able to repeatedly solubilize HABO. The shape of the carrier was selected bearing in mind previous experience with P-solubilizing fungi (Vassilev et al. 1997). The optimal inoculum size was determined simultaneously optimizing the number (amount) of the PUF particles (Table 2) using as a criterion the biomass passively immobilized in the carrier during one single cycle of 240 h.

**Table 2:** Effect of inoculum size on *P. indica* biomass immobilized on different amounts of 0.5 cm^3^ foam cubes

PUF (g flask^−1^)	Immobilized biomass (mg flask^−1^)			
	Inoculum size (ml spore/mycelium suspension)			
	0.5	1.0	2.0	5.0
0.2	210	365	433	454*
0.5	377	515	619	623
1.0	400	561	640	650

Values are means of three replicates ± standard deviation

* Standard errors are smaller than 5% and are not shown

Increasing the amount of PUF particles and inoculum size resulted in increase in the immobilized biomass. The highest dry biomass weight of 0.64–0.65 g flask^−1^ was obtained at 2.0 and 5.0 ml inoculum size and amount of 1.0 g PUF particles. It should be noted that the difference between different treatments was significant in case of 0.2 g PUF flask^−1^ and 0.5 and 1.0 g PUF flask^−1^. However, in case of maximum particles/immobilized biomass a whole embedded mass of particles-fungal mycelium was formed which provoked transfer difficulties and made no sense of the immobilization procedure as found in other similar studies (Vassilev et al. 1997). A similar pattern was observed at 0.5 g PUF flask^−1^ when groups of several particles formed whole units when the inoculum size was higher than 1.0 ml. On the contrary, at 0.2 g PUF flask^−1^, lower concentration of inoculum led to low rate of immobilization. It should be noted that the mode of inoculation by agar cubes or spore suspension did not affected the results. Therefore, based on these series of experiments, small HABO particle size (20 mesh), 0.5 g PUF flask^−1^ and 1.0 ml inoculum size were selected for the next experiment aimed at HABO solubilization by immobilized *P. indica* in conditions of repeated-batch process.

### HABO solubilization by immobilized *P. indica*

The immobilized fungus was able to solubilize HABO when employed in repeated-batch fermentation process in presence of 1.0 and 3.0 g insoluble phosphate per litre (Table 3). The concentration of soluble P increased during the experiment with 1.0 g HABO l^−1^ and reached 40.8 mg l^−1^ at the end of the 5th batch cycle with the corresponding productivity of 0.34 mg soluble P l^−1^ h^−1^. A higher initial amount of HABO resulted in two times higher level of soluble P concentration observed even at the end of the first batch cycle.

**Table 3:** P-solubilizing activity of PUF-immobilized *P. indica* in repeated-batch fermentation in the presence of HABO (1.0 and 3.0 g l^−1^)

No. batch	1.0 g l^−1^ HABO 3.0 g l^−1^			
	P soluble (mg l^−1^)	Productivity (mg l^−1^ h^−1^)	P soluble (mg l^−1^)	Productivity (mg l^−1^ h^−1^)
1	31.6 ± 0.6	0.26	62 ± 2.2	0.52
2	35.1 ± 1.1	0.29	81 ± 1.6	0.68
3	39.0 ± 0.9	0.32	95 ± 3.6	0.79
4	41.3 ± 1.0	0.34	106 ± 1.9	0.88
5	40.8 ± 0.7	0.34	120 ± 4.1	1.0

Values are means of three replicates ± standard deviation

Further improvement of the productivity was achieved with each batch cycle and the highest values of the analysed parameters (120 mg soluble P and 1.0 mg soluble P l^−1^ h^−1^) were reached at the 5th batch. The final biomass concentration reached 623 and 748 mg flask^−1^ in treatments with 1.0 and 3.0 g HABO l^−1^. It is also interesting to note that while the increase of soluble P between the first and last batch was 22.6% at 1.0 g HABO l^−1^, the same parameter at 3.0 g HABO l^−1^ was 93%.

Therefore, we could attribute the higher biomass growth of *P. indica* to the presence of soluble P in the production medium and, simultaneously, the consumption of the nitrogen source (NH_4_NO_3_) additionally maintained the conditions necessary for the HABO solubilization. The pH ranged from 4.75–4.38 to 4.05–3.92 in the treatments with 1.0 and 3.0 g l^−1^ HABO, respectively, measured at the first and last batch cycles.

### Soil–plant experiment

The effect of introduction of fermentation broth obtained after the repeated-batch process by *P. indica* in the presence or not of HABO into soil–plant system was investigated (Table 4). The addition of filter sterilized fermentation liquid of a process with *P. indica* without addition of phosphate led to significant increase of the studied parameters.

**Table 4:** Effect of the different *P. indica* fermentation liquid products on *T. repens* growth parameters

Treatment	Shoot DW (mg pot^−1^)	Shoot P (mg g^−1^ Shoot DW)	Root DW (mg pot^−1^)
C	10 ± 0.5 c	0.6 ± 0.04 c	0.7 ± 0.02 c
C+	12 ± 0.4 c	0.8 ± 0.04 c	0.7 ± 0.02 c
−P	22.9 ± 0.8 b	1.7 ± 0.57 b	1.7 ± 0.5 b
+P	48.7 ± 1.1 a	5.1 ± 0.11 a	4.3 ± 0.2 a

Values are means of four replicates ± standard deviation. Means sharing a letter are not significantly different (Tukey test, P < 0.05)

Plant shoot dry weight, root dry weight, and P shoot content were maximally increased by 2.2, 2.4, and 2.8 times, respectively, compared to the control treatment that received dH_2_O. Statistically similar results were obtained in the treatment supplemented with half-strength culture production medium. However, the treatment of soil—*T. repens* with filter-sterilized fermentation broth enriched with solubilized phosphate derived from HABO resulted in higher plant growth promoting effect. This treatment registered the highest (5.1 mg g^−1^ DW) P plant uptake amongst all another treatments. Microscopic observations confirmed that all plants contained no fungal structures.

## Discussion

Plant beneficial microorganisms produced in fermentation conditions, further formulated as bio-inoculants and applied in soil–plant systems, play an important role in sustainable agriculture by improving soil fertility and crop productivity (Bashan et al. 2014; Malusa and Vassilev 2014). The main points for the development of a commercial bio-inoculant include isolation, selection and characterization of suitable microorganism, large-scale production of biomass/spores, formulation, and testing in soil–plant systems (Vassilev et al. 2016). One of the important constrains for the large application of plant beneficial microorganisms is the fact that very few products are found to be promising (Bashan et al. 2014). This is particularly true for arbuscular mycorrhizal fungi (Faye et al. 2013), which, in addition, are difficult to be produced without host plant (Vassilev et al. 2005). During the last years, mycorrhizal-like fungi like *P. indica*, a symbiont root endophytic fungus that infests roots of a broad range of mono- and di-cotyledonous plants (Varma et al. 1999), are actively studied as they can be easily grown in axenic cultures on various complex and minimal substrates. Different techniques were studied to formulate *P. indica* including the production of cell-free products such as culture filtrates (Bagde et al. 2011; Kumar et al. 2012), The efficacy of P-enriched filtrates was recently reported for other fungi (Mendes et al. 2017). In this work however, the filtrate was produced applying the immobilized-cell technology that was widely used to study soil microorganisms (Vassilev et al. 2001). Moreover, the final product was enriched by soluble phosphate solubilized along the repeated-batch process. The results of this study are not surprising. Freely suspended mycelium of *P. indica* was recently reported to solubilize calcium phosphate and rock phosphate in fermentation processes in shake-flasks in Pikovskaya medium (Ngwene et al. 2016). However, this is the first report on solubilization of insoluble inorganic phosphate (animal bonechar) by immobilized *P. indica*. The reticulated structure of the polyurethane foam particles seems an ideal biomass carrier, as proposed originally by Atkinson et al. (1980). In this work, it efficiently enabled entrapment of biomass as reported for other, including P-solubilizing, fungal microorganisms (Vassilev et al. 1997, 2012). In the repeated batch process, we found no lag time for P solubilization at the first moments of each batch, indicating that the immobilized cells were metabolically active throughout the experiment. By this reason, we can explain the effectiveness of the shorter batch cycles and the enhancement of the volumetric productivity with each batch. HABO was recently reported as an excellent P-bearing source with a number of advantages compared to the finite, non-renewable rock phosphate (Vassilev et al. 2013). Its microbially based solubilization was also proved possible employing organic-acid producing filamentous fungi (Vassilev et al. 2012, 2013).

The significance of our research is in developing a simple method of filtrate production based on immobilized *P. indica* employed in repeated batch fermentation process in the presence of insoluble phosphate. Thus, the final liquid will contain all fito-stimulating metabolites released by the fungus and soluble phosphate.

In conclusion, the results successfully demonstrated the production of phosphate-enriched fermentation liquid by immobilized *P. indica*. By introducing this liquid into soil–plant system, improved plant growth and plant P content were registered in the test plant. The use of immobilized-based technology in producing a liquid, plant-stimulating biofertilizer offers novel opportunities of employment of *P. indica* in sustainable crop production. When assessing the results of this study, we should also note some important points such as the effect of different (growth and production) media, positive effect of immobilization (enhanced metabolic activity; lack of lag-phase and share stress, etc.), consumption of part of the soluble P, and inclusion of a part of HABO particles into the immobilized particles. Further studies should be performed to increase the productivity of the immobilized system and to prove the efficacy of the resulting product with other plant.

## Abbreviations

HABO: hydroxyapatite of animal bones origin
DW: dry weight
PUF: polyurethane foam
